# 6-Thioguanine: Antimitotic Effect on Human Lymphocytes in vitro Prevented by Adenine

**DOI:** 10.1038/bjc.1971.15

**Published:** 1971-03

**Authors:** D. J. Price, J. Timson

## Abstract

The action of 6-thioguanine on the division of PHA-stimulated human lymphocytes *in vitro* has been investigated and shown to have a definite antimitotic effect which is prevented by adenine but not by guanine or hypoxanthine. It is suggested that in this test system 6-TG exerts its main inhibitory action on phosphoribosylpyrophosphate amidotransferase.


### 6-THIOGUANINE:ANTIMITOTIC EFFECT ON HUMAN LYMPHOCYTES *IN VITRO* PREVENTED BY ADENINE

D. J. PRICE AND J. TIMSON

*From the University Department of Medical Genetics, The Royal Infirmary, Manchester M13 9WL*

Received for publication October 26, 1970

**SUMMARY.—The action of 6-thioguanine on the division of PHA-stimulated human lymphocytes *in vitro* has been investigated and shown to have a definite antimitotic effect which is prevented by adenine but not by guanine or hypoxanthine. It is suggested that in this test system 6-TG exerts its main inhibitory action on phosphoribosylpyrophosphate amidotransferase.**

THE guanine analogue, 6-thioguanine (6-TG) is an antimetabolite which has been shown to have growth inhibitory activity in a number of systems particularly cancer cells. It causes complete regression of established sarcoma 180 tumours in mice (Adams and Bowman, 1963) and in the concentration range of 0·1–0·01 mg./ml. it causes 50 % inhibition of growth of cultured human epidermoid carcinoma (KB) cells (Eagle and Foley, 1958). 6-TG has also been used therapeutically in man, particularly in the treatment of acute leukaemia in children (Burchenal *et al.*, 1956) and in chronic myeloid leukaemia. In order to obtain some insight into the possible mode of its action it was decided to investigate the effect of 6-TG on phytohaemagglutinin (PHA)-stimulated normal human lymphocytes *in vitro*.

### MATERIALS AND METHODS

Blood samples were obtained from healthy volunteers and lymphocyte cultures were set up as follows. About 20 ml. of venous blood was mixed immediately with anticoagulant (heparin in dextran) in a sterile container. The erythrocytes were allowed to settle at 37° C. and the supernatant plasma and leucocytes were drawn off. 1·5 ml. of the cell plasma suspension was made up to 10 ml. with TC199 culture medium (Glaxo) and two drops of reconstituted PHA P (Difco) were added to each culture which was then incubated at 37° C. for 72 hours. At the end of this period, 0·2 ml. of " Colcemid " (demecolcine) at a concentration of 1 mg. in 100 ml. of TC199 was added. The cultures were incubated for a further 2 hours. The cells were then spun down, the supernatant discarded and the cells resuspended in hypotonic saline for 15 minutes at 37° C. After this time, they were fixed in two changes of acetic alcohol (1 part glacial acetic acid : 3 parts ethanol) and resuspended in 45 % acetic acid to give a translucent suspension. The cells were then spread on to cold slides, air dried and stained with 10 % Giemsa buffered at pH 6·4.

The following experiments were done. In the first, the experimental cultures were exposed to varying concentrations of 6-TG throughout the 72-hour period. In subsequent experiments, equimolecular concentrations of 6-TG and either guanine, hypoxanthine, or adenine were administered simultaneously at the start of the 72-hour period. Each series of experiments was performed in triplicate. Control cultures treated in the same way, except that no 6-TG, guanine, hypoxanthine, or adenine was added, were set up at the same time.

One thousand cells per culture were examined and the number of cells in mitosis noted. The averaged results for each series of experiments are given, expressed as a percentage of the control values.

### RESULTS

The results given in Table I show that at concentrations of $10^{-3}$M and above 6-TG completely prevents mitosis and appears to kill many of the cells since morphological transformation in response to PHA is markedly reduced. Between concentrations of $10^{-6}$M and $10^{-4}$M, 6-TG caused inhibition of mitosis but had little effect on the transformation rate. Neither guanine, hypoxanthine nor adenine is able to remove the effect of 6-TG at $10^{-2}$M and $10^{-3}$M. Addition of guanine enhances the antimitotic activity of 6-TG and hypoxanthine has little effect on its action. However, between concentrations of $10^{-6}$M and $10^{-4}$M adenine protects the cells against this effect so that the mitotic index (number of mitoses/1000 cells) remains essentially the same as that of the control.

TABLE I.—*The Effect of Purines on the Antimitotic Activity of 6-thioguanine*

| Molar concentration of each added purine | 6-TG alone: No. of mitoses per 1000 cells | % control | 6-TG and guanine: No. of mitoses per 1000 cells | % control | 6-TG and hypoxanthine: No. of mitoses per 1000 cells | % control | 6-TG and adenine: No. of mitoses per 1000 cells | % control |
|---|---|---|---|---|---|---|---|---|
| $10^{-2}$ | 0 | 0 | 0 | 0 | 0 | 0 | 0 | 0 |
| $10^{-3}$ | 0 | 0 | 1 | 1·8 | 3 | 14·8 | 1 | 3·5 |
| $10^{-4}$ | 4·3 | 23·9 | 1 | 1·8 | 6·7 | 33 | 26·5 | 93 |
| $10^{-5}$ | 4 | 22·2 | 6 | 10·8 | 7 | 34·5 | 26·5 | 93 |
| $10^{-6}$ | 12 | 66·7 | — | — | 15·7 | 77·3 | 38 | 133·3 |
| 0 (control) | 18 | 100 | 55·5 | 100 | 20·3 | 100 | 28·5 | 100 |

### DISCUSSION

In the cell, 6-TG is converted to its active metabolite 6-thioguanylic acid (6-thio GMP) by the action of guanine-hypoxanthine phosphoribosyltransferase on 5-phosphoribosyl-l-pyrophosphate (PRPP) and 6-TG (Brockman, 1963). In this form it has been shown to act at a number of stages in the purine biosynthetic pathway. McCollister *et al.* (1964) have shown that it exerts a pseudofeedback effect in inhibiting the action of the enzyme phosphoribosylpyrophosphate amidotransferase from pigeon liver. This enzyme catalyses the formation of 5′-phosphoribosyl-l-amine from PRPP. Hampton (1963) with *Aerobacter aerogenes* and Meich *et al.* (1967) with sarcoma 180 ascites cells have shown that 6-thio GMP inhibits the enzyme inosine 5′-phosphate dehydrogenase (IMP dehydrogenase) which catalyses the conversion of inosinic acid (IMP) to xanthylic acid (XMP). Meich *et al.* (1967) also report the inhibitory action of 6-thio GMP on ATP : GMP phosphotransferase isolated from hog brain tissues, which converts guanylic acid (GMP) to guanosine 5′-diphosphate (GDP). Any of these effects would limit the amount of guanine nucleotides formed both to act as coenzymes and in nucleic acid synthesis, thus resulting in inhibition of nucleic acid synthesis with consequent inhibition in mitotic activity. Le Page (1960) has reported the incorporation of small amounts of 6-thio GMP into nucleic acids of tumour cells. Furthermore, cells which incorporate 6-TG into their DNA remain viable but do not replicate their DNA (Le Page and Jones, 1961; Le Page, 1963).

Clearly the antimitotic effect of 6-TG on cultured lymphocytes could be explained by one or more of the above effects. However the results given in Table I suggest the possibility that 6-TG may be exerting its prime effect in this system early in the purine biosynthetic pathway. Purine bases can be converted to their corresponding ribonucleotides by reacting with PRPP. For this reaction, both guanine and hypoxanthine require guanine-hypoxanthine phosphoribosyltransferase, the same enzyme which converts 6-TG to 6-thio GMP. Since neither guanine nor hypoxanthine can reverse the antimitotic activity of 6-TG it suggests that 6-T G competes successfully with these purines for this enzyme, so that the guanine and hypoxanthine are not in fact utilized by the cell. The increase in antimitotic activity when guanine is administered with 6-TG is probably due to a cytotoxic effect since transformation was also slightly lowered.

Adenine requires a different enzyme—adenine phosphoribosyltransferase—to convert it to the ribonucleotide adenylic acid (AMP) and may therefore compete with the 6-TG for the available PRPP. This would result in a lowering of the active amount of 6-thio GMP formed and hence account for the prevention of antimitotic activity.

It is also possible that some of the AMP formed in this way could be deaminated to IMP and hence lead to the formation of XMP and GMP. If the main inhibitory action of 6-TG was on phosphoribosylpyrophosphate amidotransferase, then the activity of any 6-thio GMP which was formed could be bypassed and a supply of purine ribonucleotides ensured so that mitotic activity could proceed normally.

Thus 6-TG seems to react preferentially with guanine-hypoxanthine phosphoribosyltransferase in the presence of equal concentrations of either guanine or hypoxanthine but adenine appears to preferentially utilize the available PRPP in human lymphocytes when 6-TG and adenine are both present. This might be expected in view of the role which ATP plays in the energy relationships of the cell.

We wish to thank the Smith Kline and French Foundation for financial support and Mrs. Barbara Haynes for technical assistance.

### REFERENCES

Adams, D. H. and Bowman, B. M.—(1963) *Cancer Res.*, **23**, 883.

Brockman, R. W.—(1963) *Adv. Cancer Res.*, **7**, 129.

Burchenal, J. H., Murphy, M. L. and Tan, T. C.—(1956) *Pediatrics, Springfield*, **18**, 643.

Eagle, H. and Foley, G. E.—(1958) *Cancer Res.*, **18**, 1017.

Hampton, A.—(1963) *J. biol. Chem.*, **238**, 3068.

Le Page, G. A.—(1960) *Cancer Res.*, **20**, 403.—(1963) *Cancer Res.*, **23**, 1202.

Le Page, G. A. and Jones, H.—(1961) *Cancer Res.*, **21**, 1590.

McCollister, R. J., Gilbert, W. R., Ashton, D. H. and Wyngaarden, J. B.—(1964) *J. biol. Chem.*, **239**, 1560.

Meich, R. P., Anderson, J. H. and Sartorelli, A. C.—(1967) *Biochem. Pharmac.*, **16**, 2222.


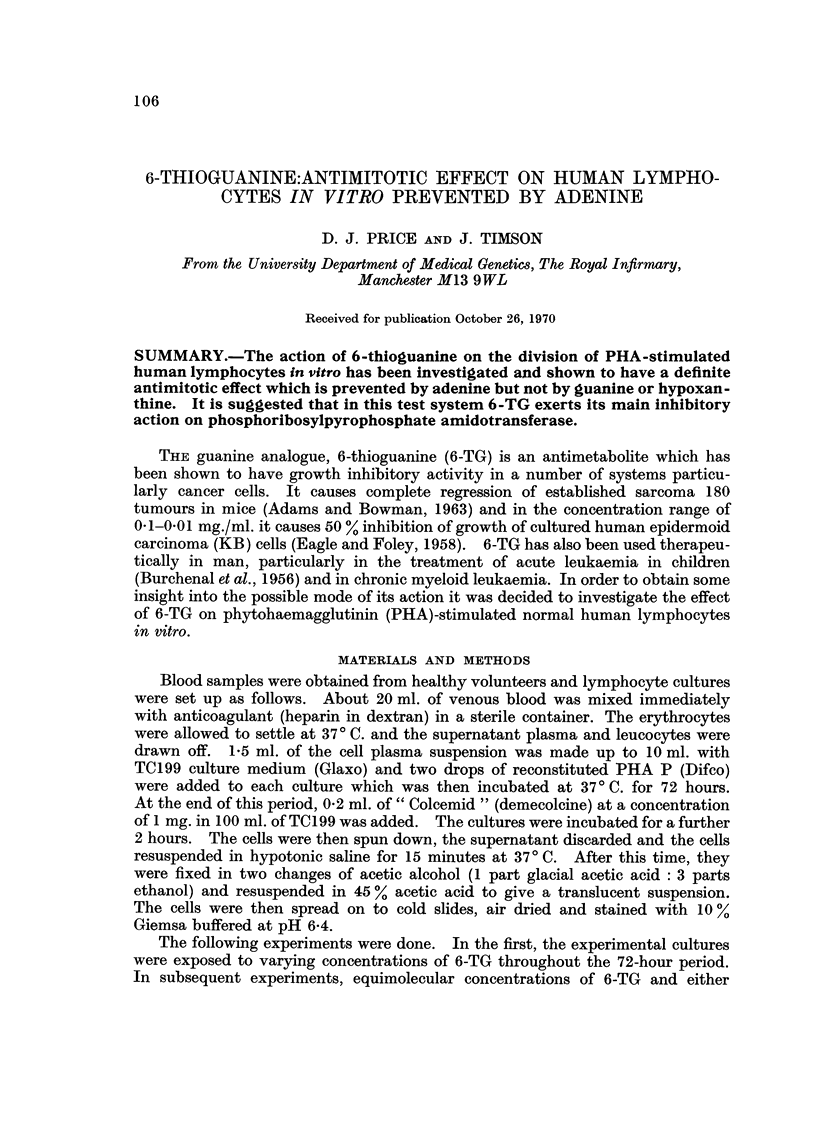

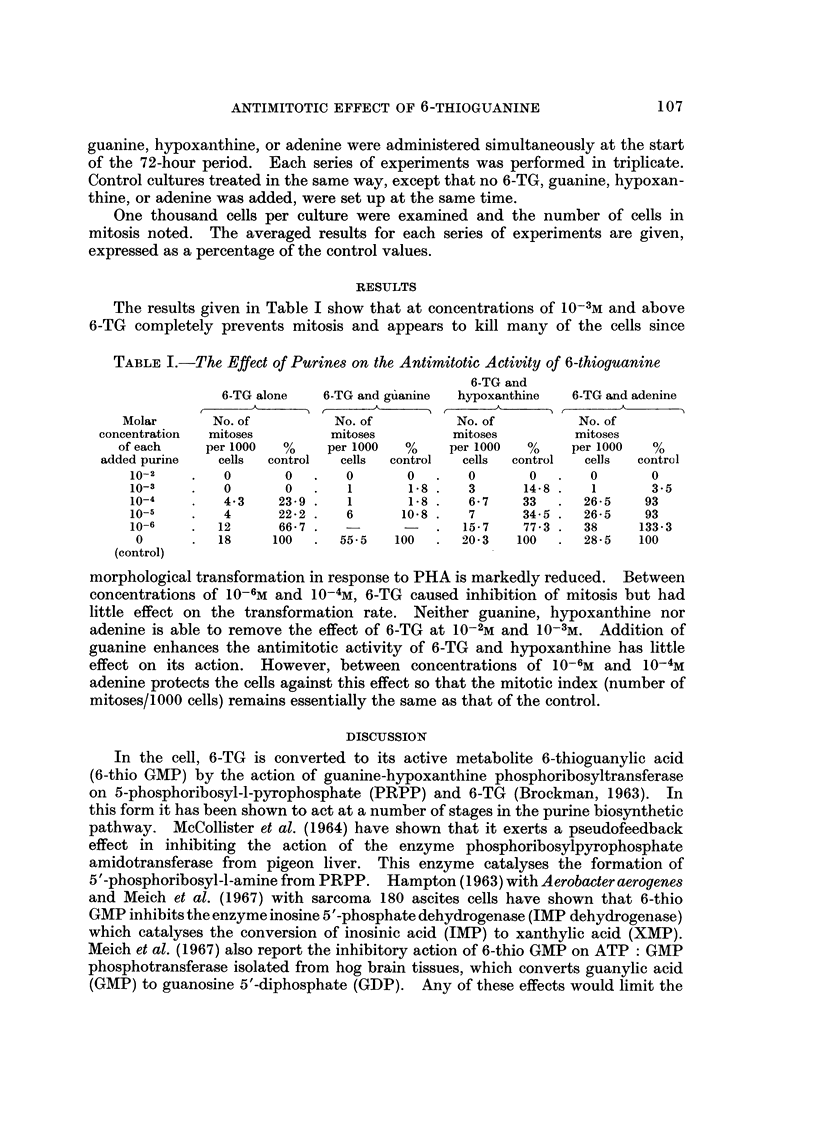

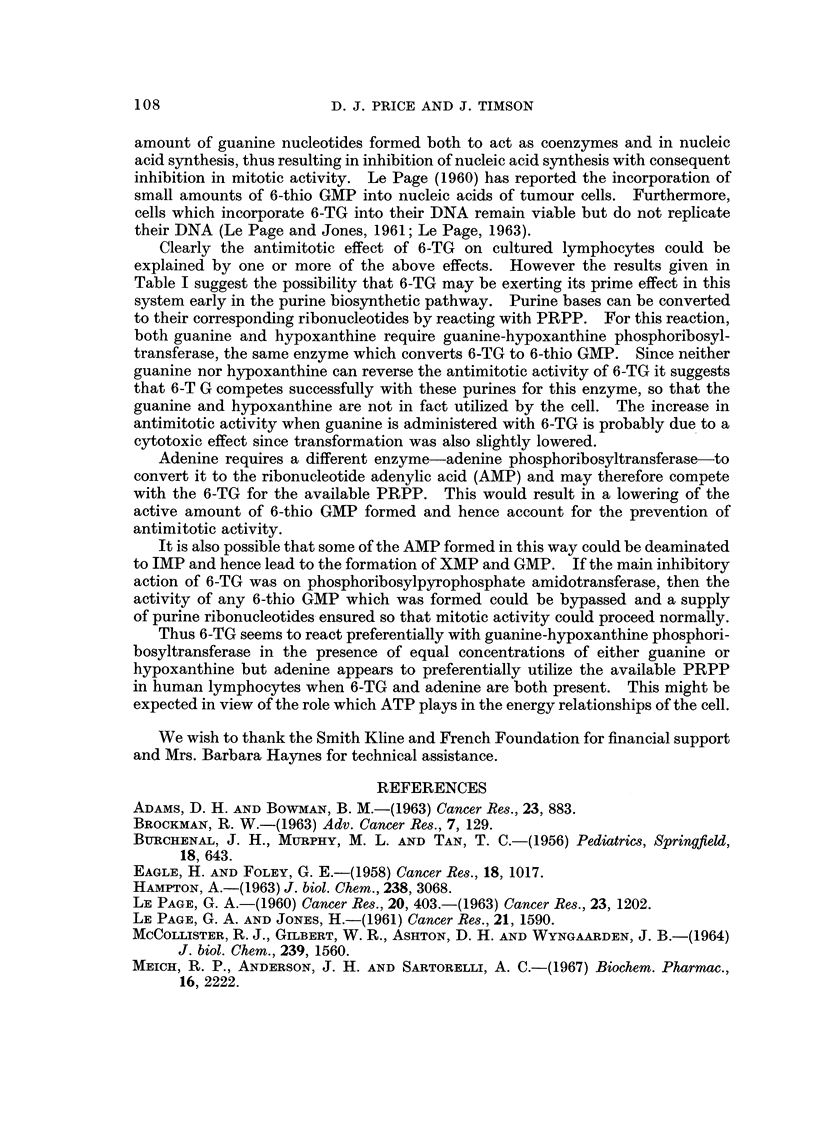


## References

[OCR_00177] BROCKMAN R. W. (1963). MECHANISMS OF RESISTANCE TO ANTICANCER AGENTS.. Adv Cancer Res.

[OCR_00179] BURCHENAL J. H., MURPHY M. L., TAN C. T. (1956). Treatment of acute leukemia.. Pediatrics.

[OCR_00183] EAGLE H., FOLEY G. E. (1958). Cytotoxicity in human cell cultures as a primary screen for the detection of anti-tumor agents.. Cancer Res.

[OCR_00184] HAMPTON A. (1963). REACTIONS OF RIBONUCLEOTIDE DERIVATIVES OF PURINE ANALOGUES AT THE CATALYTIC SITE OF INOSINE 5'-PHOSPHATE DEHYDROGENASE.. J Biol Chem.

[OCR_00189] MCCOLLISTER R. J., GILBERT W. R., ASHTON D. M., WYNGAARDEN J. B. (1964). PSEUDOFEEDBACK INHIBITION OF PURINE SYNTHESIS BY 6-MERCAPTOPURINE RIBONUCLEOTIDE AND OTHER PURINE ANALOGUES.. J Biol Chem.

[OCR_00193] Miech R. P., Parks R. E., Anderson J. H., Sartorelli A. C. (1967). An hypothesis on the mechanism of action of 6-thioguanine.. Biochem Pharmacol.

